# Supplementary material for: Active Sampling Techniques for Two-Spot Cotton Leafhopper (Amrasca biguttula Ishida) (Hemiptera: Cicadellidae) in Fields with High and Low Populations
Source: Insects. 2025 Dec 3;16(12):1226. doi: 10.3390/insects16121226 (PMC12734267; doi:10.3390/insects16121226)
Supplement: Supplementary file 1 [file insects-16-01226-s001.zip › insects-3972671-supplementary.pdf]

Table S1. Comparison of the Generalized Linear Mixed Model (GLMM) using negative binomial distribution and the Analysis of Variance (ANOVA) using log (x + 1) transformation analyses of total number of leafhoppers (adults + nymphs).

**Model Effects**		**GLMM Negative Binomial Distribution**		**ANOVA log (x + 1) Transformation**	
Response Variable	Df	Chisq	Prob>Chisq	F Ratio	Prob>F
Sample Method	7	124.1636	2.2e-16	11.5028	<.0001
Stage	1	9.1446	0.002495	5.7091	0.0229
Worker	4	51.6344	1.645e-10	5.8017	0.0013
Sample Method*Stage	7	82.4935	4.267e-15	4.9003	0.0008
Sample Method*Worker	28	97.7592	1.165e-09	1.2657	0.2586

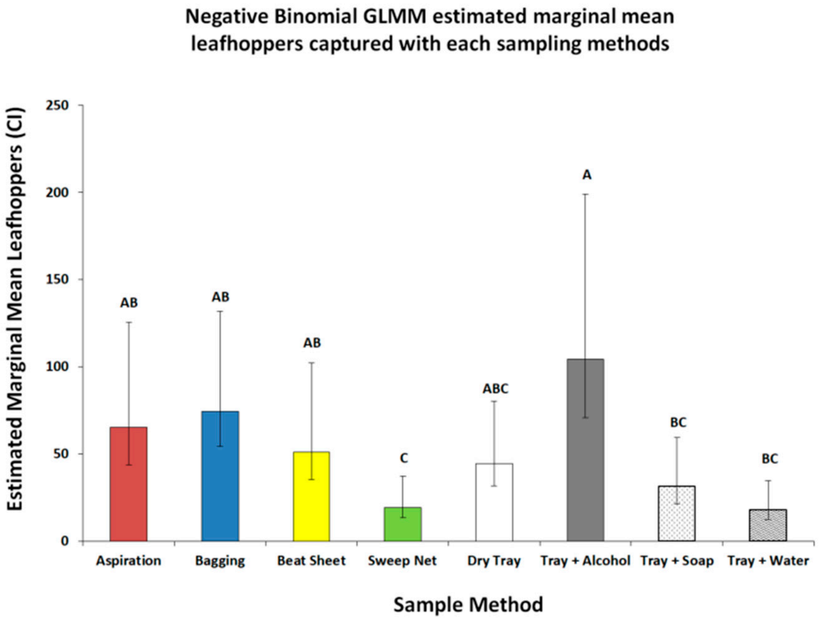

Figure S1. The estimated marginal mean (± Confidence Interval) number of leafhoppers (sum of adults and nymphs) per person for each sampling method from a highly infested okra field. Treatments sharing a letter were considered statistically similar, while those with distinct letters were considered significantly different.
